# Supplementary material for: Mechanisms governing the pioneering and redistribution capabilities of the non-classical pioneer PU.1
Source: Nat Commun. 2020 Jan 21;11:402. doi: 10.1038/s41467-019-13960-2 (PMC6972792; doi:10.1038/s41467-019-13960-2)
Supplement: Supplementary file 7 — Source data [file 41467_2019_13960_MOESM7_ESM.zip › Source_Data/Figure6/Figure6B_MotifScanOutput/ETS-specific/homerResults/motif37.similar.html]

motif37

## Information for motif37

T
A
G
C
T
C
G
A
T
C
G
A
T
C
G
A
T
G
C
A
G
C
A
T
C
A
T
G
C
A
T
G
T
G
A
C
A
C
T
G
A
T
C
G
A
G
T
C
  
Reverse Opposite:  

T
C
A
G
T
A
G
C
T
G
A
C
A
C
T
G
G
T
A
C
G
T
A
C
C
G
T
A
A
C
G
T
A
G
C
T
A
G
C
T
A
C
G
T
A
T
C
G
  

|  |  |
| --- | --- |
| p-value: | 1e-23 |
| log p-value: | -5.420e+01 |
| Information Content per bp: | 1.699 |
| Number of Target Sequences with motif | 196.0 |
| Percentage of Target Sequences with motif | 2.56% |
| Number of Background Sequences with motif | 406.6 |
| Percentage of Background Sequences with motif | 1.01% |
| Average Position of motif in Targets | 104.9 +/- 56.3bp |
| Average Position of motif in Background | 102.0 +/- 62.1bp |
| Strand Bias (log2 ratio + to - strand density) | 0.0 |
| Multiplicity (# of sites on avg that occur together) | 1.06 |
| Motif File: | file (matrix) reverse opposite |

### Similar de novo motifs found

|  |  |  |  |  |  |  |  |
| --- | --- | --- | --- | --- | --- | --- | --- |
| Rank | Match Score | Redundant Motif | P-value | log P-value | % of Targets | % of Background | Motif file |
| 1 | 0.928 | T A G C A C T G A T G C T G A C C T A G A G T C A G T C C G T A A G C T A G T C A C G T A C G T A C T G T C A G | 1e-22 | -52.921861 | 1.55% | 0.44% | motif file (matrix) |
| 2 | 0.878 | C T G A C G T A A T C G C T G A A C G T A C T G C T A G G A T C A T C G C A T G A G T C | 1e-22 | -51.141381 | 1.76% | 0.57% | motif file (matrix) |
| 3 | 0.947 | G A C T T G A C C T G A G T C A A C T G C T G A A G C T A T C G A C T G A G T C A T C G C A T G T G A C | 1e-21 | -48.616677 | 2.01% | 0.73% | motif file (matrix) |
| 4 | 0.817 | C T G A C G T A A T C G C G T A A C G T A T C G T A C G A G T C A T C G C A T G | 1e-17 | -39.164363 | 1.87% | 0.74% | motif file (matrix) |
